# Comment on “The efficiency of risedronate in reducing bone resorption after total hip arthroplasty: a meta-analysis of randomized control trials at a minimum of 6 months’ follow-up”

**DOI:** 10.1186/s13018-022-03118-0

**Published:** 2022-04-12

**Authors:** M. M. Kai Huang, M. M. Gang Wang, M. D. Yi Zeng

**Affiliations:** 1 Department of Orthopedics, Orthopedic Research Institute and National Clinical Research Center for Geriatrics, West China Hospital, Sichuan University, 37 Guoxue Road, Chengdu, 610041 Sichuan Province People’s Republic of China; 2 Laboratory of Stem Cell and Tissue Engineering, Orthopedic Research Institute, State Key Laboratory of Biotherapy and Cancer Center, West China Hospital, Sichuan University and Collaborative Innovation Center of Biotherapy, Chengdu, 610041 People’s Republic of China

Dear Editor,

Total hip arthroplasty (THA) has become a widely accepted and reliable surgical option for end-stage hip osteoarthritis. Nevertheless, periprosthetic bone resorption after THA is inevitable, which may predispose to aseptic loosening, periprosthetic fractures, and subsequentially increasing challenges at revision surgery. Nowadays, risedronate has been used in the clinic as an attempt to prevent bone resorption and reduce postoperative complications. However, its efficacy still remains controversial. Yang et al. [1] performed a meta-analysis which concluded that oral risedronate could significantly reduce periprosthetic bone resorption around an uncemented femoral stem (Gruen zones 1, 2, 3, 6, and 7) up to 6 months after THA without increased risk of adverse events. We appreciate the authors’ work in this field; however, some issues in the article that may nullify the conclusion should not be ignored.

Firstly, this is a meta-analysis of randomized control trials (RCTs), while the authors used Methodological Index for Non-Randomized Studies (MINORS) scale to assess the methodological quality of the included studies in methods section, which was obviously incorrect. Besides, the authors declared that 6-month cutoff was used for statistical analysis because all RCTs were at a minimum of 6-month follow-up. However, to our knowledge, one of the included studies [2] had only reported the final results at 4 years in the article without 6-month data. Furthermore, we noticed that two studies [2, 3] in the meta-analysis came from the same cohort. Thus, extracting duplicate data from both literatures for analysis would be more likely to increase the bias and lead to an incorrect conclusion.

Indeed, as a new generation of bisphosphonates (BPs), risedronate can effectively inhibit osteoclast and promote mineralization. In the early postoperative period, the use of risedronate can have significant short-term prevention of periprosthetic bone resorption. However, Muren et al. [2] found that risedronate did not prevent the development of bone resorption at 4 years after THA, which was opposite to the outcome itself at 1 year [3]. And the obviously declining trend of risedronate’s efficacy on periprosthetic bone mineral density (BMD) was similar to other BPs in RCTs with medium-term follow-up, such as pamidronate (5-year follow-up) [4] and alendronate (5-year follow-up) [5]. As a result, with the continuous action of stress shielding and the discontinuation of risedronate, its efficacy against bone resorption still has a great dispute in the medium or long-term follow-up. Additionally, Aro et al. [6] demonstrated that zoledronate had long-lasting protective efficacy on periprosthetic bone resorption but did not enhance the initial femoral stem stability, which means the relevance between increased periprosthetic BMD and the benefit of final prognosis is still inconclusive. Last but not least, several recent retrospective studies had reported that the long-term intake of BPs was associated with atypical periprosthetic fractures [7, 8], which even made the safety of risedronate face challenges.

Given all that, in addition to focusing on periprosthetic BMD, future larger clinical trials with a longer duration of follow-up are supposed to pay more attention to the efficacy of risedronate on clinically relevant endpoints such as aseptic loosening, periprosthetic fracture, and revision arthroplasty. And beyond that, the optimal dose and length of risedronate treatment should also be a key topic in future researches.

## Data Availability

Not applicable.

## Abbreviations

THA: Total hip arthroplasty
RCTs: Randomized control trials
MINORS: Methodological Index for Non-Randomized Studies
BPs: Bisphosphonates
BMD: Bone mineral density
